# Celebrating eighty years of radionuclide therapy and the work of Saul Hertz

**DOI:** 10.1002/acm2.13175

**Published:** 2021-02-03

**Authors:** Frederic H. Fahey, Frederick D. Grant

**Affiliations:** ^1^ Department of Radiology Boston Children’s Hospital Harvard Medical School Boston MA USA; ^2^ Department of Radiology Children’s Hospital of Philadelphia University of Pennsylvania School of Medicine Philadelphia PA USA

**Keywords:** history, radioiodine therapy, thyroid

## Abstract

March 2021 will mark the eightieth anniversary of targeted radionuclide therapy, recognizing the first use of radioactive iodine to treat thyroid disease by Dr. Saul Hertz on March 31, 1941. The breakthrough of Dr. Hertz and collaborator physicist Arthur Roberts was made possible by rapid developments in the fields of physics and medicine in the early twentieth century. Although diseases of the thyroid gland had been described for centuries, the role of iodine in thyroid physiology had been elucidated only in the prior few decades. After the discovery of radioactivity by Henri Becquerel in 1897, rapid advancements in the field, including artificial production of radioactive isotopes, were made in the subsequent decades. Finally, the diagnostic and therapeutic use of radioactive iodine was based on the tracer principal that was developed by George de Hevesy. In the context of these advancements, Hertz was able to conceive the potential of using of radioactive iodine to treat thyroid diseases. Working with Dr. Roberts, he obtained the experimental data and implemented it in the clinical setting. Radioiodine therapy continues to be a mainstay of therapy for hyperthyroidism and thyroid cancer. However, Hertz struggled to gain recognition for his accomplishments and to continue his work and, with his early death in 1950, his contributions have often been overlooked until recently. The work of Hertz and others provided a foundation for the introduction of other radionuclide therapies and for the development of the concept of theranostics.

## 1 Introduction

On March 31, 1941, Elizabeth D, a patient referred to Dr. Saul Hertz at the Massachusetts General Hospital for management of hyperthyroidism, was administered 2.1 mCi of a ^130^I/^131^I mixture. Thus, she became the first patient with thyroid disease to be treated with radioiodine. She received a second administration of 1.3 mCi on April 16, 1941. Dr. Hertz subsequently administered radioactive iodine therapy to another 28 patients over the next two years and demonstrated a high rate of successful therapy of hyperthyroidism.

Only five years earlier, on November 12, 1936, Dr. Hertz, the head of the Thyroid Unit at Massachusetts General Hospital (MGH), attended a faculty luncheon at Harvard Medical School. Karl Compton, President of the Massachusetts Institute of Technology (MIT), was the guest speaker on the topic of “What Physics Can Do for Medicine and Biology.” Incidentally, he was the older brother of Arthur Compton who would win the Noble Prize in Physics for his discovery and characterization of what would come to be known as “Compton scattering.” Also attending the luncheon was J. Howard Means, Chief of Medicine at MGH and founder of the Thyroid Unit and Robley Evans, chief of the Radioactivity Laboratory at MIT. At the end of Dr. Compton’s presentation, Dr. Hertz spontaneously asked what would be considered the seminal question that led to targeted radionuclide therapy and eventually to the concept of “theranostics.” He asked Dr. Compton “Can iodine be made radioactive?”

Leading up to Dr. Hertz’s question, four discoveries and advancements first needed to occur: the understanding of the role of iodine in thyroid function, the discovery of radioactivity, the development of the tracer principle, and the artificial production of radioactive elements.^1^

## 2 Iodine and the Thyroid

The condition known as “goiter”, that is, pathological enlargement of the thyroid gland, was recognized in many parts of the ancient world including China, India, and the Mediterranean. The Romans attributed the high incidence of goiter in people living in the Alps to the drinking of water from mountain streams. The Renaissance physician, philosopher, and alchemist Paracelsus and others also recognized an increased prevalence of congenital goiter and mental retardation in the mountainous regions of Europe,^2^ but treatment of goiter was limited and typically not successful. However, some early experiences suggested that treatment with sea water, sponge, or seaweed (all now known to be iodine‐rich) might have some beneficial effect.^3^, ^4^ In the 18^th^ and 19^th^ centuries, several physicians, including Robert Graves, published reports describing what is now called Graves’ Disease, with an association of goiter, exophthalmos, and cardiac palpitations.^5^ In 1811, Bernard Courtois isolated iodine from the ashes of seaweed.^6^ Soon after, Gay‐Lussac confirmed that iodine was a new element, and derived the name from the Greek ioeides, meaning “violet colored”. Within a few years (1816), Proust treated patients with goiter with iodine. In 1851, Adolphe Chatin reported an increased prevalence of goiter in populations with low iodine intake.^7^ In the 1890s, Eugen Baumann demonstrated that the thyroid contained “thyroiodine”, a proteinaceous substance containing 10‐15% iodine, and that the administration of thyroiodine could reverse myxedema (severe hypothyroidism).^3^, ^4^ Around the same time, Adolf Oswald was able to isolate thyroglobulin. In 1919, Edward Kendall at the Mayo Clinic isolated crystalline thyroxine.^8^ During this time Charles Mayo, who introduced the term “hyperthyroidism”, worked to develop improved surgical management of thyroid diseases. For patients with hyperthyroidism, this included pre‐operative medical management of Graves’ disease with administration of large doses of non‐radioactive iodine under the supervision of Henry Plummer.^9^ Thus, by 1920, the association between iodine and the thyroid was clearly established, and the use of non‐radioactive iodine to treat thyroid diseases was becoming more widespread.

## 3 X‐rays and Radioactivity

In November 1895, while performing a series of experiments with cathode‐ray tubes, Wilhelm Röntgen discovered a previously undescribed radiation that he called “x‐rays.” His findings were published by the end of that year and, within 6 months, x‐rays were being used for diagnostic medical imaging. For his discovery, Röntgen was awarded the first Nobel Prize in Physics in 1901. A few months after Röntgen’s discovery, Henri Becquerel, who had been studying possible photoluminescence of uranium salts sought to determine if x‐rays were being emitted from the uranium salts. He demonstrated that similar rays were being emitted from the uranium, even without prior excitation by sunlight, and, thus, represented an inherent property of the material. Marie Curie, a graduate student at the time, coined the term “radioactivity” to describe this phenomenon.

Over the next several years, Marie Curie and her husband, Pierre Curie, were able to isolate and identify two additional and previously unknown radioactive elements, radium and polonium. For their work in discovering radioactivity and discovering these radioactive elements, Becquerel and the Curies were awarded the Nobel Prize in Physics in 1903. In 1911, Marie Curie also was awarded the Nobel Prize in Chemistry, making her the only person to ever receive the Nobel Prize in the fields of both physics and chemistry. The potential therapeutic action of radioactivity was appreciated very early on, and, in 1909, Marie Curie and the University of Paris established the Radium Institute, later renamed the Curie Institute, to investigate the biological and medical effects of radioactivity.^10^, ^11^

Although it was now clear that there existed radioactive elements such as radium and polonium, it was later shown that there were radioactive isotopes of some elements that also had nonradioactive isotopes. For example, Georg de Hevesy demonstrated in 1913 that a radioactive species resulting from the radioactive decay of radium and referred to as “radium‐D” was actually a radioisotope of lead, ^210^Pb. However, the question remained as to whether these radioisotopes behaved in a biologically identical manner to the nonradioactive isotopes of the same element. In 1923, George de Hevesy reported that, in plants, the absorption and translocation of radioactive lead was similar to that of non‐radioactive lead and demonstrated the potential of radioactive indicators to study living organisms.^12^ In 1943, Dr. de Hevesy would be awarded the Nobel Prize in Physiology and Medicine for what is now referred to as the “tracer principle,” i.e. a small amount of a radioactive indicator can be used to characterize the physiological behavior of its non‐radioactive congener. The tracer principle is the foundation of diagnostic and therapeutic nuclear medicine, as well as other approaches to molecular imaging.

In the 1930s, Marie Curie’s daughter, Irène Joliot Curie, and her husband, Frederic Joliot Curie, a former graduate student of Marie, were investigating the effects of bombarding a *number of substances* with alpha particles. Chadwick and Anderson recently had demonstrated that such techniques led to the emission of new particles and the discovery of the neutron and positron, respectively.^13^, ^14^ In 1934, the Joliot‐Curies bombarded aluminum with alpha particles, and found that neutrons were promptly emitted, while positrons were emitted not promptly but with an exponential delay. This and subsequent experiments confirmed that their experiments had produced “artificial” or man‐made radioactivity:^27^Al_13_ + ^4^α_2_ → ^30^P_15_ + ^1^n_0_ → ^30^Si_14_ + β^+^ with the decay of ^30^P to ^30^Si and the release of a positron occurring with a 2.5 min half‐life. They reported their findings later in 1934,^15^ and, within a few months, many other research groups from around the world, including Enrico Fermi and the Berkeley group led by Ernest Lawrence, also were generating artificially produced radioisotopes. For this work, the Joliot‐Curies were awarded the 1935 Nobel Prize in Chemistry.

## 4 The Luncheon and the Question

**Fig. 1:**
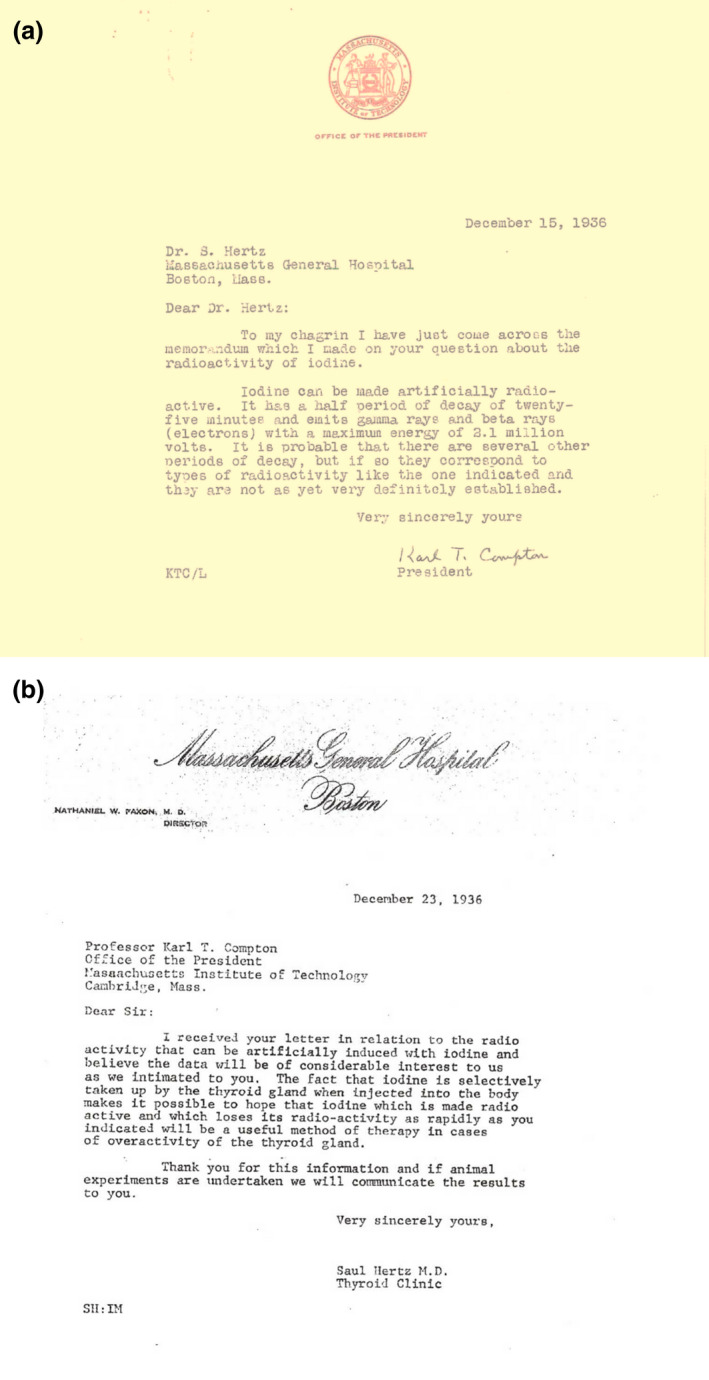
(a) Follow‐up letter sent by Dr. Compton to Dr. Hertz, acknowledging Hertz’s question about radioactive iodine posed at the Harvard faculty luncheon. Compton responded that iodine can be made radioactive emitting gamma rays and beta particles with half‐life of 25 min. (b) Dr. Hertz’s response to Compton’s letter indicating that radioactive iodine may provide “a useful method of therapy in cases of overactivity of the thyroid gland.”

This brings us to the Harvard Medical School faculty luncheon on November 12, 1936. At this point, the importance of iodine to thyroid function is known and iodine is being used routinely to treat thyroid diseases. Radioactivity has been discovered and had been the subject of intense research for nearly forty years, and the tracer principle was utilized as a tool to study biologic processes. In the two years prior to this luncheon, radioactive isotopes of many elements had been produced artificially. Dr. Hertz’s boss Dr. Means had investigated the treatment of toxic goiter with externally applied x‐rays. So Dr. Hertz was primed to ask his question, “Can iodine be made radioactive?”. Dr. Means noted in a later application to The Markle Foundation, "...it at once occurred to Hertz that we might make use of them (radioactive iodine isotopes) to solve a problem we were already working on."^16^ In subsequent correspondence (Fig. 1) between Compton and Hertz, Compton informed Hertz that a radioactive isotope of iodine (now known to be ^128^I, Table 1) was available, and Hertz clearly stated his hypothesis that radioactive iodine might be used to treat thyroid diseases.

**Table 1:** Isotopes of iodine, including stable ^127^I which occurs naturally with 100% abundance, and longer lived radioactive isotopes.

Isotope	Half‐life	Decay	Energy	Production
^123^I	13.2 h	EC	159 keV (83%)	^123^I‐NaI
^124^I	4.2 d	b^+^ g	1550 + keV 511 keV	^124^I‐NaI
^125^I	57.4 d	EC	35 keV (100%)	^124^Xe(n,g)^125^I
^127^I			STABLE	
^128^I	25 min	b^‐^	440‐1400 keV	
^130^I	12.4 h	b^‐^ g	620‐1000 keV	^130^I‐NaI
^131^I	8.1 d	b^‐^ g	250‐810 keV 364 keV (81%)	^130^Te(n,g)^131^I ^235^U fission

The iodine radioisotope mentioned in the December 1936 letter from Professor Karl Compton to Dr. Saul Hertz is ^128^I, which, at the time, could be produced in low abundances by neutron activation, using a Ra‐Be neutron source. Dr. Hertz’s first patient was treated with a mixture of ^130^I (90%) and ^131^I (10%).

## 5 Preliminary Experiments

**Fig. 2:**
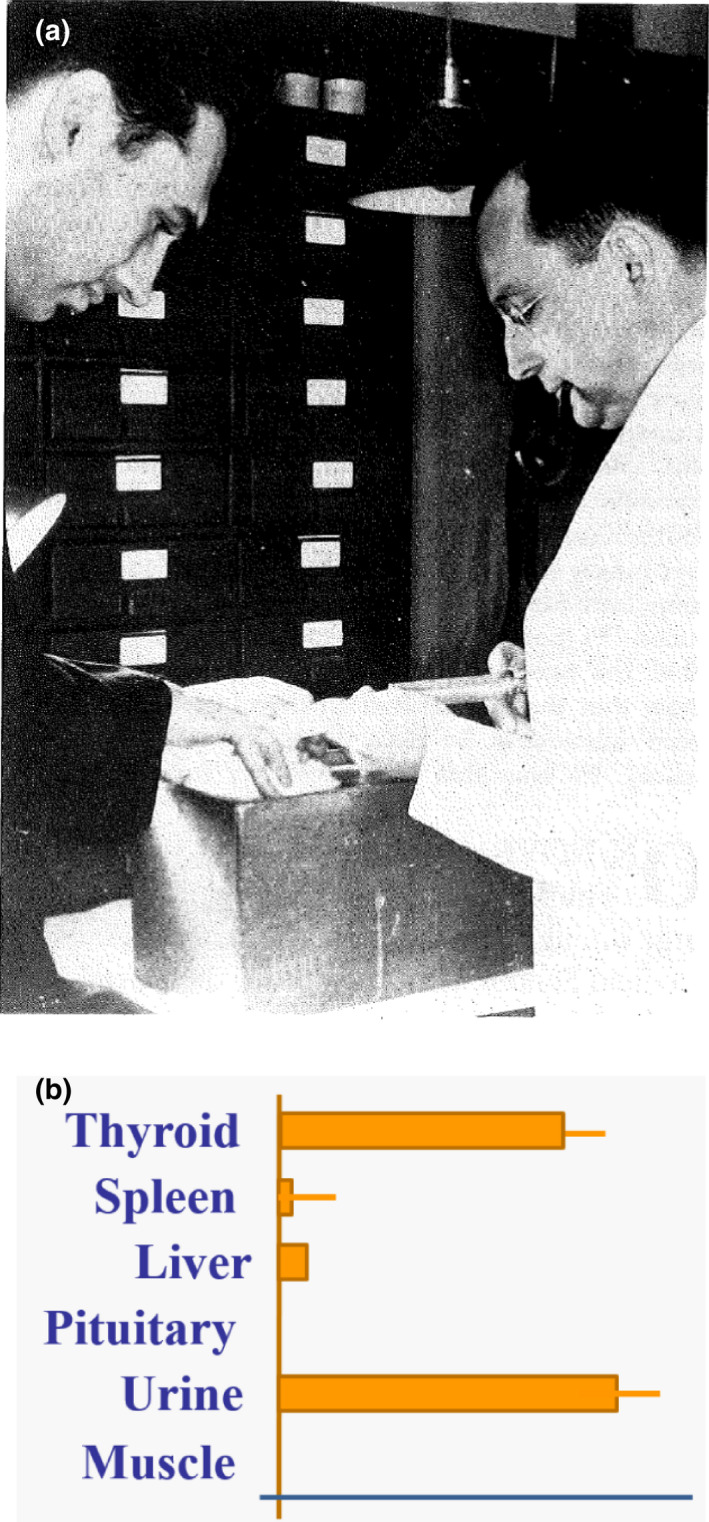
(a) Drs. Hertz and Roberts (right and left, respectively) perform an ear vein injection into a rabbit for their biodistribution experiments. In contrast with how these studies might be conducted now, it is noted that Drs. Hertz and Roberts are not wearing gloves and Dr. Hertz is smoking a pipe. (b) Early results of biodistribution studies show that radioiodine is concentrated in the thyroid, as reported at the meeting of the Society of Experimental Biology and Medicine in 1938.

As a result of this exchange of letters, a collaboration was established between investigators at MGH and at MIT. Robley Evans PhD, director of the MIT Radioactivity Center, had recently hired Arthur Roberts who would work directly with Dr. Hertz on the essential experiments leading up to treatment of the first patient. Funding was made available from the Harvard Medical School for preliminary animal studies. Radioactive iodine (^128^I) was produced by neutron activation, using a Ra‐Be neutron source, in a manner first developed by Enrico Fermi.^17^, ^18^ Utilizing the resulting small amounts of ^128^I, *in vivo* experiments in rabbits confirmed that radioactive iodine was concentrated in the thyroid gland (Fig. 2).^19^ Even at this early stage, Hertz postulated that radioactive iodine might also be used to treat thyroid cancer.

Although small quantities of ^128^I (25‐min half‐life) were satisfactory for studies of iodine biodistribution, Hertz and Roberts realized that larger quantities of longer‐lived iodine radioisotopes would be necessary for the therapeutic use of radioactive iodine. ^130^I and ^131^I were the most appropriate candidates (Table 1). Ernest Lawrence and his Berkeley colleagues were using accelerating charged particles for high‐energy physics experiments and had developed the cyclotron accelerator to support this research. For this work, Lawrence was awarded the 1939 Nobel Prize in Physics. After the Joliot‐Curie publication describing the artificial creation of radioactive elements, the Berkley group quickly realized that the cyclotron also could be used to produce artificial radioactivity. With the successful use of the cyclotron by the Berkeley group and others to create radioactive isotopes, the MIT team along with Drs. Means and Hertz made plans to construct a cyclotron on the MIT campus “primarily for medical purposes.” They approached Markel Foundation in New York for funding and received a $30,000 grant for the purchase and installation of a cyclotron at MIT. Installation commenced in the fall of 1938 and a cyclotron with a 42” magnet became operational in November 1940. In its initial operations, a mixture of ^130^I (90%) and ^131^I (10%) was produced.

## 6 The First Patients

Let us return to the case of Elizabeth D, a patient referred to Dr. Hertz for management of hyperthyroidism. At the time of her referral, she had an elevated basal metabolic rate (BMR + 30), which was in keeping with the increased metabolic activity associated with hyperthyroidism. She became the first patient treated with radioiodine for thyroid disease on March 31, 1941, using the ^130^I/^131^I mixture produced by the newly operational cyclotron at MIT. She received two radioiodine doses; 2.1 mCi on March 31 and an additional 1.3 mCi on April 16. Follow‐up evaluation showed that her BMR decreased from + 30 to −7 after radioiodine therapy. As the standard of care for hyperthyroidism remained thyroidectomy, the patient underwent surgical thyroidectomy and surgical pathology showed thyroid involution. Based on clinical evaluation and these findings, Dr. Hertz classified her as “not cured”, although there appears to have been some response to the radioactive iodine treatment.

Over the next few years, Hertz administered radioactive iodine to 29 patients with the clinical diagnosis of hyperthyroidism. Two pages of Dr. Hertz’s patient log are shown in Fig. 2. Of these first 29 patients, 20 were considered cured of their disease as a result of radioiodine treatment and 9 were considered not cured, although post‐thyroidectomy demonstrated thyroid involution in the six of these “non‐cured” patients. The initial experience of these treatments was reported at the 34^th^ Annual Meeting of the American Society for Clinical Investigation held in May 1942.^20^ It is notable that, at the same meeting, John Lawrence and Joseph Hamilton from Berkeley reported on their use of radioiodine to treat patients with hyperthyroidism starting in October 1941.

At the height of World War II, Dr. Hertz decided to join the military and was granted a military leave of absence to serve as a Commander in the US Navy. Upon the departure of Dr. Hertz, Earle Chapman was named Director of the MGH Thyroid Unit by Dr. Means. Dr. Chapman had been working primarily as a clinician associated with the Unit and was familiar with the work of Hertz and Roberts.

## 7 After World War II

At the conclusion of the war, Dr. Hertz returned to Boston, but was not welcomed back to his previous position at the MGH. He instead joined the medical staff at the Beth Israel Hospital in Boston, where he would continue to investigate the use of radioiodine to treat benign and malignant thyroid disease, with the support of funding from the U.S. Navy . In the spring of 1946, it was brought to Dr. Hertz’s attention by Morris Fishbein, the editor of the Journal of the American Medical Association (JAMA), that Drs. Chapman and Evans had submitted a manuscript describing their use of radioactive iodine to treat 22 patients with hyperthyroidism. This work had been performed during Dr. Hertz’s absence for military service, and the manuscript did not recognize the prior work of Hertz and Roberts. At Fishbein’s invitation, Hertz and Roberts submitted a manuscript describing their earlier use of radioiodine to treat 29 patients. The two manuscripts were published in the same issue of JAMA, with the Hertz and Roberts article given precedence.^21^, ^22^ Interestingly, although both papers reported on the same topic and originated at the same institution, there were no authors in common between the two articles.

Later the same year, in December 1946, Dr. Samuel Seidlin and colleagues at Montefiore Hospital in New York City published in JAMA a report describing the use of radioactive iodine to treat 23 patients with metastatic thyroid cancer.^23^ Drs. Seidlin and Hertz had discussed this initial work on using radioiodine for the treatment of malignant disease on several occasions. Inspired, in part, by Dr. Hertz’s work and with his consultation, the Manhattan Project announced the availability of radioisotopes for civilian scientific and medical use in June of 1946.^24^ In addition, Drs. Hertz and Seidlin established the Radio Isotope Research Institute in Boston with the purpose of developing applications for nuclear fission products and other radionuclides for the treatment of thyroid disease including cancer as well as other malignancies.

Over the next few years, Dr. Hertz further developed his research laboratory at the Beth Israel Hospital until opening a radioactive isotope research division at the Massachusetts Women’s Hospital in Boston in September 1949.^25^ Unfortunately, he had little time to advance his studies of radionuclide therapy (using radioactive iodine and radioactive phosphorus) and radiation detection before his sudden death because of a heart attack at age 45 in 1950.

## 8 Legacy of Saul Hertz

In the 80 years since Dr. Hertz treated Elizabeth D, an untold number of patients around the world have been treated with radioiodine for both benign and malignant thyroid disease. Although our understanding of radiobiology and dosimetry has advanced, patients continue to be treated in much the same way as Dr. Hertz treated his patients. During subsequent decades, radionuclide therapy was introduced for other diseases, but these other therapies never achieved the success or acceptance that radioiodine had for treatment of thyroid diseases.

In the past decade, the use of targeted radionuclide therapy has expanded dramatically with the promise of being a mainstay for the treatment of neuroendocrine tumors, and prostate cancer. In recent years, the term theranostics, a portmanteau of therapeutics and diagnostics, has been used to describe the use of similar (and usually radioactive) drugs to both image and treat disease. As the promise of theranostics develops, it is important to remember Saul Hertz and to have the courage to ask the right question, to understand the import of the moment, and to not let an opportunity to develop a breakthrough therapy pass by. For these reasons, we continue to honor and celebrate the pioneering, visionary work of Dr. Saul Hertz.

9

**Fig. 3:**
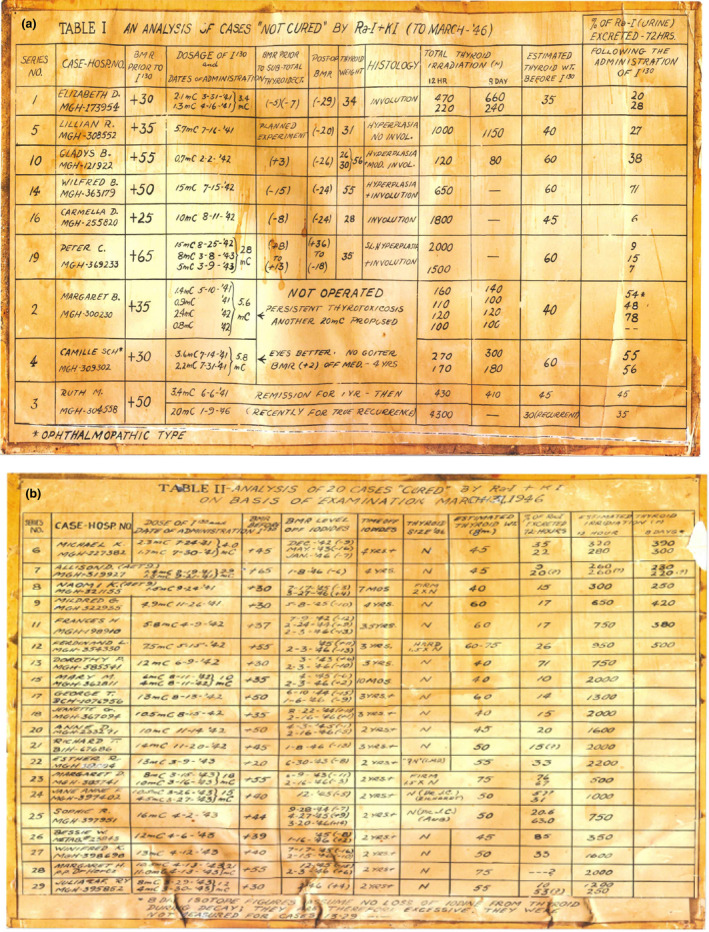
Two pages from Dr. Hertz’s log of the first 29 treated with radioiodine between 1941 and 1943. Patients were divided into two groups: (a) those that were deemed “NOT CURED” (9 patients) and (b) those deemed “CURED” (20 patients). One of these patients initially treated in June 1941, received a follow‐up treatment for a disease recurrence in 1946. Although Elizabeth D. is listed as Patient 1 at the top of the “NOT CURED” list, her BMR was reduced to −7 prior to thyroidectomy and histology showed evidence of involution, suggesting atleast a partial response to therapy.
